# No association detected between posterior tibial slope, static anterior tibial translation and medial meniscus repair failure after anterior cruciate ligament reconstruction

**DOI:** 10.1002/jeo2.70715

**Published:** 2026-04-23

**Authors:** David Mazy, Nicolas Cance, Lucia Angelelli, Tomas Pineda, Michael James Dan, David Henri Dejour

**Affiliations:** ^1^ Orthopedic Surgery Department, Lyon Ortho Clinic Clinique de la Sauvegarde Lyon France; ^2^ Clinica Ortopedica e Traumatologica 2 IRCCS Istituto Ortopedico Rizzoli Bologna Italy; ^3^ Facultad de Medicina, Hospital del Trabajador Universidad Andrés Bello Santiago Chile; ^4^ Facultad de Medicina, Hospital el Carmen Universidad Finis Terrae Santiago Chile; ^5^ East Coast Athletic Orthopaedics Macquarie and Lingard Hospital Merewether New South Wales Australia

**Keywords:** anterior cruciate ligament reconstruction, medial meniscus, meniscus repair, posterior tibial slope, static anterior tibial translation

## Abstract

**Purpose:**

This study aimed to evaluate the influence of the posterior tibial slope (PTS) and static anterior tibial translation (SATT) on the success of medial meniscus (MM) repair performed concomitantly with anterior cruciate ligament reconstruction (ACLR).

**Methods:**

In this retrospective study, all patients who underwent primary ACLR using hamstring autograft combined with isolated MM repair between January 2014 and December 2017, and a minimum follow‐up of 6 years, were included. Patients who had undergone meniscectomy or lateral meniscus repair were excluded. Demographic data, PTS, SATT, dynamic anterior tibial translation (DATT), the need for reoperation (at the same location) for MM repair failure (MMRF), and time to failure were recorded. Comparative analyses were performed using thresholds of 12° for PTS and 5 mm for SATT. Univariate logistic regression analyses were used to identify independent risk factors for MMRF.

**Results:**

Among the 148 patients included, 14 (9.4%) experienced a MMRF at a mean of 24 ± 16 months post‐operatively (range 7–60 months). Twenty‐eight percent of patients had undergone concomitant lateral extra‐articular tenodesis (LET). There were no significant differences between the MMRF and non‐failure groups in terms of age, sex, presence of LET, PTS, SATT or DATT. Patients with PTS ≥ 12° (odds ratio, 2.9; 95% confidence interval, 0.8–11.6; *p* = 0.11) or SATT ≥ 5 mm did not demonstrate a higher rate of MMRF. No variable from the univariate analysis met the criteria for inclusion in the multivariate analysis. Limited number of MMRF events increase potential risk of type II error.

**Conclusion:**

No statistically significant association was detected between PTS, SATT, DATT, age or the presence of LET and MMRF after hamstring ACLR. However, larger studies are needed, particularly in high‐slope subgroups. Increased PTS or SATT alone should not discourage MM repair in the setting of ACLR.

**Level of Evidence:**

Level III, retrospective case–control study.

**Abbreviations**
ACL: anterior cruciate ligament
ACLR: anterior cruciate ligament reconstruction
DATT: dynamic anterior tibial translation
LET: lateral extra‐articular tenodesis
LM: lateral meniscus
MM: medial meniscus
MMRF: medial meniscus repair failure
PTS: posterior tibial slope
ROM: range of motion
SATT: static anterior tibial translation

## INTRODUCTION

Anterior cruciate ligament (ACL) injuries are rarely an isolated injury. There is a high incidence of injury to either or both the medial and lateral meniscus, with a greater incidence in delayed ACL reconstruction (ACLR) [12, 16]. Meniscal preservation is essential whenever possible, with reported success rates ranging from 70% to 94% in well‐selected indications [11, 20, 23, 35]. Medial meniscus (MM) repair carries a higher risk of failure compared with lateral meniscus (LM) repair ^42^. Reported failure rates range from 20% to 25% for MM and 10% to 15% for LM, reflecting the greater biomechanical demands on the MM due to its reduced mobility and role as a secondary anteroposterior stabiliser [13, 42]. Outcomes of meniscal repair are improved when performed in combination with ACLR [32, 37, 41]. Prognostic factors include vascular zone, tissue quality, chronicity and complexity of the tear, and knee stability [22, 31, 33]. Failure rates increase significantly up to 40% in unstable knees, emphasising again the importance of concomitant anterior cruciate ligament reconstruction (ACLR) [9, 36]. Coronal alignment, particularly varus deformity, negatively impacts the healing of chronic degenerative medial root tears [5]. Sagittal knee bony morphology is also critical. Increased posterior tibial slope (PTS) and static anterior tibial translation (SATT) are established risk factors for ACLR graft rupture [24, 34], as they increase anterior tibial translation and graft stress [6, 14]. High PTS has been identified as a risk factor for secondary MM tears in ACL‐deficient knees [21]. Lateral PTS is associated with LM root tears, while medial PTS correlates with ramp lesions in acute ACL injuries and with medial root tears in degenerative knees [10, 19, 25]. SATT also increases with MM tears in the setting of ACL injury [7]. Persistence of increased anteroposterior shear forces in the presence of a high PTS after ACLR may also influence graft remodelling and contribute to secondary medial meniscal lesions [40].

Given there is a correlation between PTS, SATT and MM injury, the relationship between PTS, SATT, and MM repair failure (MMRF) using all‐inside cerclage technique after ACLR needs to be explored. The objective of this study was to assess the impact of PTS and SATT on MMRF after ACLR. We hypothesised that increased PTS and SATT place greater biomechanical stress on the repaired MM, resulting in higher failure rates.

## METHODS

### Study design

A retrospective analysis was performed on a consecutive series of ACLR performed in a sports knee referral centre between January 2014 and December 2017. Inclusion criteria included primary single‐bundle ACLR with pedicular hamstrings autograft with MM repair, age ≥ 14 years old, and a minimum follow‐up of 6 years. Exclusion criteria included associated meniscectomy or LM repair, revision surgery, additional associated procedures (e.g., deflexion or coronal plane osteotomy, cartilage surgery), multi‐ligamentous knee injuries and patients with neurological and rheumatological disorders. Patients with a concomitant ACL graft rupture were excluded because it's difficult to know if the failure of the meniscal repair occurs during or after the graft rupture. This study focused exclusively on MM repairs without any concomitant procedure on the LM, to minimise potential confounding factors introduced by additional surgical interventions. During the follow‐up period, only nine concomitant ACL graft ruptures occurred, representing a small but distinct subgroup.

### Surgical technique

All patients underwent Outside‐In ACLR using a hamstring tendon autograft. The semitendinosus and gracilis tendons were harvested with an open stripper. Femoral and tibial fixation were achieved with bioabsorbable interference screws (Ligafix; SBM). The femoral tunnel was positioned centrally to replicate both bundles through a single‐bundle reconstruction, performed with an outside‐in technique.

The MM was repaired using an all‐inside technique with one or two sutures, depending on tear size, using a cerclage/'hay bale' configuration [15]. This technique was selected to provide stable fixation for peripheral longitudinal and ramp lesions while minimising the risk of iatrogenic cartilage injury. All repairs were performed after arthroscopic confirmation of tear instability and adequate vascular zone viability (red‐red or red‐white zone). The use of a uniform all‐inside repair technique across all cases was intended to minimise technical variability and strengthen the internal validity of the study.

### Approach to the lateral extra‐articular tenodesis

A LET was performed in all patients younger than 18 years of age. In patients older than 18 years of age, LET was selectively performed in cases of genu recurvatum > 10°, hyperlaxity (Beighton score ≥ 4/9), and/or pivot shift Grade 2 or 3 [17]. LET was performed using the modified Lemaire technique, in which a 1 × 9 cm strip of the posterior iliotibial band was harvested, passed deep to the lateral collateral ligament and fixed with a 7 mm interference screw (Ligafix; SBM) in a femoral tunnel positioned 5 mm proximal and 5 mm posterior to the lateral collateral ligament insertion at 80° of knee flexion with neutral rotation [8].

### Post‐operative management

All patients were discharged home on the same day of the surgery. All patients followed the same rehabilitation programme, which involved isometric quadriceps exercises, passive and active range of motion (ROM) exercises (0°–90°) starting on postoperative day one. ROM was gradually advanced, so full ROM was achieved by 6 weeks. No brace was used. Weight‐bearing as tolerated with crutches was allowed for 21 days and then full weight bearing. No specific modifications to the postoperative weight‐bearing or knee flexion restriction protocols were applied for the longitudinal or ramp meniscal lesions included in this study. Only radial, root, or bucket‐handle tears followed a non‐weight‐bearing protocol with restricted knee flexion; however, these lesion types were not included in the present analysis. Return to sports was typically permitted at nine months based on functional and isokinetic test results.

### Data collection

All data were prospectively added into an institutional registry: patient age at time of surgery, gender, laterality and associated LET were recorded. In cases of MMRF, the time between surgery and revision was recorded.

### Imaging patient assessment

All patients underwent radiographic evaluation in the same institution and radiology department. Radiographs included an antero‐posterior view, and a true lateral knee radiographs in monopodal weight bearing at 20° flexion with a minimum of 15 cm of proximal tibia visible. The PTS and SATT measurements were performed using HOROs DICOM viewer software (version 3.3.6), on the pre‐operative radiographs by observers blinded to clinical outcomes. The PTS was measured using the proximal anatomical axis method [3]. PTS was measured by calculating the angle between the perpendicular to the tibial diaphysis, and the tangent to the anterior and posterior edges of the medial tibial plateau. SATT was defined as the distance between two lines parallel to the posterior tibial cortex, the first tangent to the posterior aspect of the medial tibial plateau, and the second tangent to the posterior femoral condyles [4]. Postoperative SATT was not systematically assessed and therefore was not included in the present analysis. However, ACLR has not been shown to significantly modify SATT post‐operatively [30]. Dynamic anterior tibial translation (DATT) was measured using the Telos device with an applied anterior force of 15 kg [27]. The side‐to‐side difference was recorded, and the reported DATT corresponded to the preoperative difference between limbs, with positive values indicating greater anterior translation on the ACL‐deficient side. Fifty radiographs were independently reviewed to calculate the intraclass correlation coefficient (ICC) by two examiners (orthopaedic surgeons D.M. and N.C.). Intraobserver reliability was evaluated by repeating measurements twice by one examiner (D.M.) with a 2‐week interval.

### Patient follow‐up

At a minimum of 6 years follow‐up, patients were contacted by phone and email and queried regarding any subsequent surgery for MM tear diagnosis following their primary ACLR. An MMRF was identified if a tear occurred at the same location as described in the initial ACLR operative report. Both the primary ACLR and the subsequent surgery for MM tear were performed by the same senior author (D.H.D.). MM tears included ramp or longitudinal lesions, while bucket‐handle tears, radial tears, and degenerative medial root tears were excluded. Patients were considered lost to follow‐up if no response was obtained after five phone calls and three emails over a period of 6 months. The follow‐up used is the longest possible with our database. But the majority of MMRF occur within the first two postoperative years [42].

### Subgroup analysis

Patients were separated according to a PTS threshold of 12° and an SATT threshold of 5 mm. These cut‐off values have been reported in prior studies as clinically meaningful thresholds associated with an increased risk of ACLR graft rupture [28, 34, 39]. They were selected to ensure consistency with the existing literature and to avoid the use of new thresholds. To examine the effect of LET addition; a subgroup analysis was conducted comparing those with and without LET. A comparison was also performed for the entire cohort based on gender. Additionally, the MMRF group was compared to the group without MMRF.

### Statistical analysis

Normally distributed continuous variables are reported as the mean ± standard deviation. Dichotomous variables are expressed as the number and percentage of patients. The Shapiro–Wilk normality test was utilised to assess the normal distribution of the sample. For comparing variables between groups, the chi‐square test or exact Fisher test (if categories below 5% of the population) was employed for categorical variables. Independent‐samples t‐test or Mann–Whitney *U* test was used for continuous variables, depending on the normality test results. To identify risk factors for MMRF, univariate analyses of each factor separately were used, with the threshold for multivariate logistic regression analysis set at *p* < 0.2. As none of the variables met this criterion, multivariable analysis was not performed. Odds ratios and confidence intervals were reported for the variables associated with the outcome. The ICC was calculated for PTS, SATT and DATT measurements. SPSS Statistics software (version 29.0.1.0; IBM Corp, Armonk [13], NY, USA) was used to perform these statistical analyses. Statistical significance was set to *p* < 0.05.

## RESULTS

### Population

**Figure 1:**
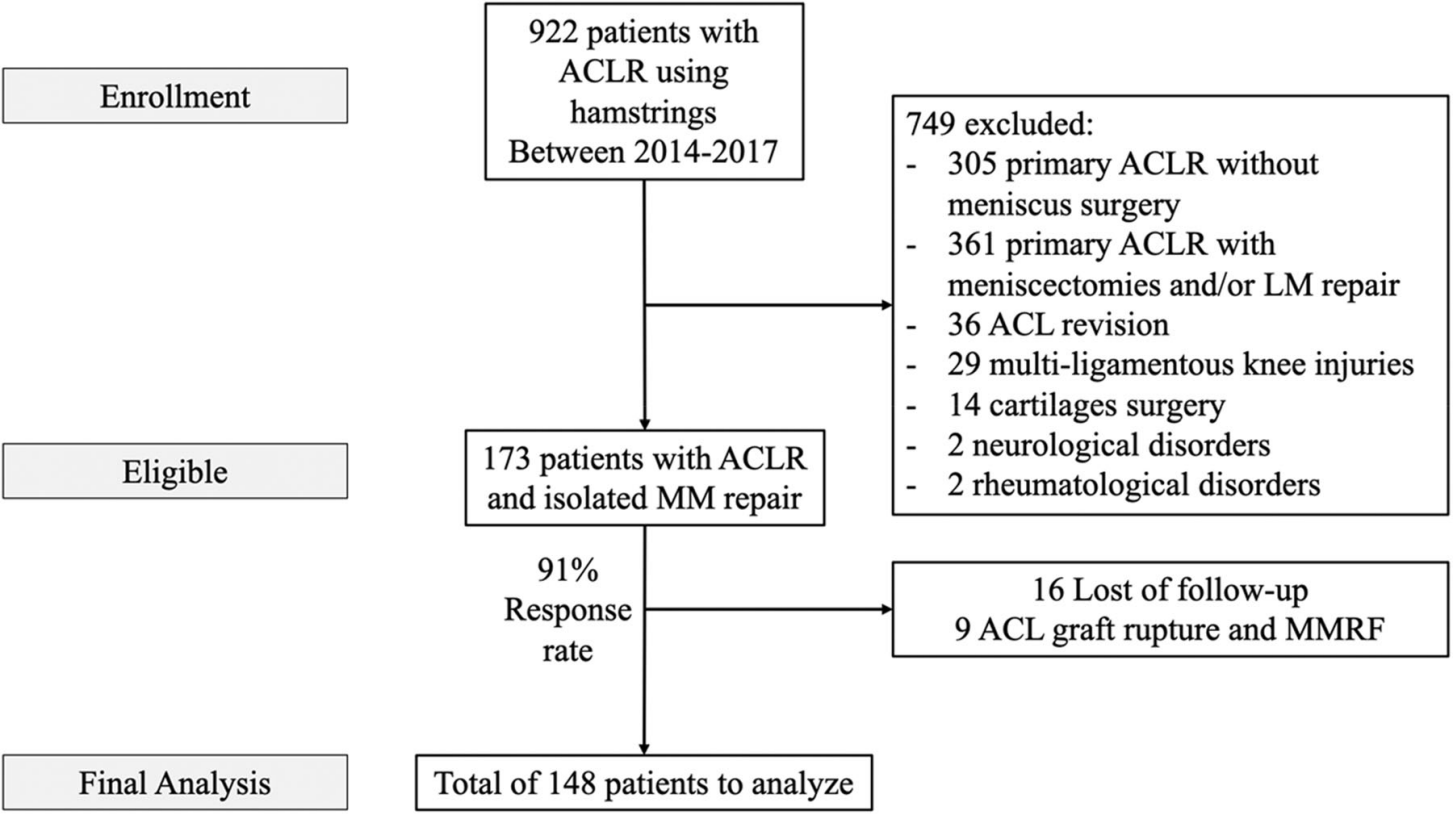
Study flowchart. ACL, anterior cruciate ligament; ACLR, anterior cruciate ligament reconstruction; LM, lateral meniscus; MM, medial meniscus.

In total, 164 patients met the inclusion criteria. Of these, 16 were lost to follow‐up, leaving 148 patients for analysis (90% response rate). The study cohort is summarised in Figure 1. Fourteen (9.4%) MMRF were recorded during the study period. The mean follow‐up was 94 months (range 72–120 months) and the mean time to MMRF was 24 ± 16 months post‐operatively (range 7–60 months). No MMRF were recorded beyond 5 years post‐operatively. Demographic data and radiographic measurements are shown in Table 1.

**Table 1:** Patients' demographics and radiographic measurements.

Demographics	Mean (*n* = 148)	SD	[Min–max]
Age (years)	29	10	[14–56]
Gender (female/male)	58/90		
Side (right/left)	78/70		
MMRF	14 (9.4%)		
LET	42 (28%)		
Radiographic measurements			
PTS (°)	9.2	2.4	[3–15]
SATT (mm)	2.7	3.4	[−4 to 12]
DATT (mm)	6.5	3.4	[0–16]

Abbreviations: DATT, dynamic anterior tibial translation; LET, lateral extra‐articular tenodesis; MMRF, medial meniscus repair failure; PTS, posterior tibial slope; SATT, static anterior tibial translation.

### Subgroups analysis

The results of group comparisons according to the PTS and SATT thresholds are presented in Tables 2 and 3.

**Table 2:** Comparison of patients using a PTS threshold of 12°.

	PTS < 12° (*n* = 124)	PTS ≥ 12°(*n* = 24)	*p*‐Value
Age (years)	29 ± 10	29 ± 11	0.899
Gender (female %)	46 (37%)	12 (50%)	0.248
Side (right %)	62 (50%)	16 (67%)	0.144
MMRF	10 (8.1%)	4 (16.7%)	0.193

Abbreviations: MMRF, medial meniscus repair failure; PTS, posterior tibial slope.

**Table 3:** Comparison of using a SATT threshold of 5 mm.

	SATT < 5 mm (*n* = 114)	SATT ≥ 5 mm (*n* = 34)	*p*‐Value
Age (years)	29 ± 10	30 ± 11	0.477
Gender (female %)	38 (33%)	13 (38%)	0.949
Side (right %)	52 (46%)	18 (53%)	0.883
MMRF	11 (9.6%)	3 (8.8%)	0.732

Abbreviations: MMRF, medial meniscus repair failure; SATT, static anterior tibial translation.

The results of group comparisons according to the presence of a MMRF and the presence of LET are presented in Tables 4 and 5.

**Table 4:** Comparison of patients with MMRF and patients without.

	No MMRF (*n* = 134)	MMRF (*n* = 14)	*p*‐Value
Age (years)	29 ± 10	28 ± 10	0.688
Gender (female %)	53 (40%)	5 (36%)	0.780
LET	38 (28%)	4 (29%)	0.987
PTS	9.2 ± 2.3	9.1 ± 2.9	0.946
SATT	3 ± 3.4	1.4 ± 3	0.104
DATT	6.4 ± 3.5	7.6 ± 3	0.213

Abbreviations: DATT, dynamic anterior tibial translation; LET, lateral extra‐articular tenodesis; MMRF, medial meniscus repair failure; PTS, posterior tibial slope; SATT, static anterior tibial translation.

**Table 5:** Comparison of patients with and without LET.

	No LET (*n* = 106)	LET (*n* = 42)	*p*‐Value
Age (years)	32 ± 10	22 ± 7	<0.001
Gender (female %)	45 (42%)	13 (31%)	0.196
PTS	9.2 ± 2.4	9.3 ± 2.3	0.799
SATT	2.8 ± 3.3	2.9 ± 3.6	0.915
DATT	6.3 ± 3.3	6.9 ± 3.7	0.320
MMRF	10 (9.4%)	4 (9.5%)	0.987

Abbreviations: DATT, dynamic anterior tibial translation; LET, lateral extra‐articular tenodesis; MMRF, medial meniscus repair failure; PTS, posterior tibial slope; SATT, static anterior tibial translation.

Three of the 20 patients (15%) younger than 18 years experienced MMRF, compared with 11 of the 128 adult patients (8.6%) (*p* = 0.363), but the analysis is limited by the small number of paediatric patients.

### Gender comparison

There was no difference in MMRF between sexes (*p* = 0.780). Age, PTS, SATT, DATT and the use of LET also showed no sex‐related differences (Table 6).

**Table 6:** Gender comparison.

	Female (*n* = 58)	Male (*n* = 90)	*p*‐Value
Age (years)	28 ± 12	29 ± 9	0.565
PTS	9.5 ± 2.2	9 ± 2.4	0.272
SATT	2.8 ± 3.2	2.8 ± 3.5	0.968
DATT	6.4 ± 3.6	6.5 ± 3.3	0.819
LET	13 (22%)	29 (32%)	0.196
MMRF	5 (8.6%)	9 (10%)	0.780

Abbreviations: DATT, dynamic anterior tibial translation; LET, lateral extra‐articular tenodesis; MMRF, medial meniscus repair failure; PTS, posterior tibial slope; SATT, static anterior tibial translation.

The univariate logistic regression analysis indicated that posterior tibial slope ≥12°, static anterior tibial translation ≥ 5 mm, age < 18 years, the presence of a LET, and gender were not statistically significantly associated with MMRF (Table 7).

**Table 7:** Univariate logistic analysis of risk factors for MMRF.

Variable	OR	95%CI	*p*‐Value
PTS ≥ 12°	2.9	0.8–11.6	0.114
SATT ≥ 5 mm	0.7	0.2–2.8	0.600
Age < 18 years	3.5	0.6–20.7	0.162
Without LET	0.8	0.2–2.9	0.610
Female sex	0.6	0.2–2.2	0.450

Abbreviations: CI, confidence interval; LET, lateral extra‐articular tenodesis; MM, medial meniscus repair failure; OR, odds ratio; PTS, posterior tibial slope; SATT, static anterior tibial translation.

There were excellent intra‐ and interobserver reliability ICCs for PTS (0.945 and 0.937, respectively), SATT (0.980 and 0.962, respectively) and DATT (0.925 and 0.910, respectively) measurements.

## DISCUSSION

The most important findings of the present study were that PTS, SATT, DATT, the absence of LET and gender were not significantly associated with an increase rate of MMRF, using a all inside suture in a cerclage/'hay bale' technique for peripheral longitudinal and ramp tears.

The mean timing to MMRF is comparable to the literature with 2.1 ± 1.6 years discovered in the study by Westermann et al. [42]. No MMRF was observed beyond 5 years post‐operatively, suggesting a time‐dependent pattern of failure. The present study found a failure rate of 9.4% with a minimum follow‐up of 6 years, coherent to Westermann et al. who reported a 10.3% reoperation rate (16/149) for MMRF without concomitant ACL graft rupture. Nepple et al. reported a 23.3% reoperation rate for isolated MM repair at 5 years [26], which the difference may be explained by the concomitant ACLR in our series as described by Wasserstein et al (9.7% reoperation rate for both MM and LM repairs combined with ACLR compared to 16.7% for meniscus repair alone) [41].

The influence of PTS on outcomes after ACLR has been widely documented, with increased slope being consistently associated with higher graft rupture rates [24, 34]. In the present study, a PTS ≥ 12° did not significantly increase the risk of MMRF. Higher medial and lateral PTS have been associated with secondary meniscal lesions on magnetic resonance imaging 1 year after ACLR [40]. One proposed mechanism is that an increased PTS augments compressive and shear forces at the posterior medial meniscus root [40]. Although the odds ratios in the present study suggested a trend toward an increased risk, the limited sample size reduced statistical power, and these findings should therefore be interpreted with caution. Clinically, this lack of association may indicate that, while PTS contributes to increased anterior tibial translation and graft loading, it does not exert the same magnitude of stress on meniscal repairs when performed concomitantly with ACLR. Increased PTS or SATT alone should not discourage MM repair in the setting of ACLR. In contrast, when ACLR is not performed concomitantly, PTS has been identified as a risk factor for secondary MM tears, highlighting its major role in anteroposterior stability in the ACL‐deficient knee [21]. Authors hypothesised that the use of the MM as a brake for anterior tibial translation increase the rate of failure by increasing the stress on the meniscus and the suture.

SATT, a parameter closely related to PTS, also showed no significant association with MMRF [4, 24]. Previous studies have identified both PTS and SATT as risk factors for ACL graft failure, but the current results suggest that these anatomical and radiographic factors may have a limited impact on the healing process of the MM [2, 24].

This supports the concept that, in the setting of ACLR, the graft acts as the primary restraint to anteroposterior tibial translation and sufficiently reduces the mechanical load on the medial meniscus to protect the repair, regardless of variations in PTS or SATT. These findings further support the systematic performance of ACLR when addressing combined ACL rupture and medial meniscus tear, to maximise the meniscal healing [41].

The classical doctrine was that for a meniscal re‐tear to occur following ACLR, the graft is inappropriately positioned or failed. DATT, measured through pre‐operative stress radiographs, was also not associated with MMRF. While increased post‐operative dynamic translation is a reliable predictor of graft failure risk and poorer knee‐related quality of life, as well as decreased function in sports, its role in meniscal repair prognosis appears minimal. It is plausible that, once the ACL is reconstructed, the meniscus is shielded from excessive anterior translation, thereby limiting its exposure to detrimental shear forces and so not correlated to the pre‐operative DATT.

Regarding the presence of LET, no difference in MMRF was observed. This finding should be interpreted with caution, as patients who underwent LET were significantly younger, reflecting a selection bias related to our institutional practice, in which all patients younger than 18 years systematically received a LET. The absence of association with MMRF reinforces the hypothesis that LET primarily acts to reduce pivot‐shift instability of the lateral compartment, with no direct association with MM repair healing [18].

In large cohorts, sex has not been shown to influence ACLR revision rates [29]. Regarding meniscus, male adolescents exhibit a higher prevalence of lateral meniscus injuries [32]. In our series, sex did not affect the rate of MMRF. One possible explanation is that differences between sexes may be more relevant for LM injuries, while MM repair outcomes appear less dependent on sex‐related factors.

Young age has also been reported as a negative prognostic factor for meniscal repair, with failure rates ranging from 17% to 28% in paediatric populations [22, 38]. In our study, no association with age was identified but it is not a paediatric population. This likely reflects the small number of adolescents included (*n* = 20, aged 14–18 years), which does not represent the larger paediatric cohorts of the aforementioned studies [22, 38].

Multiple surgical techniques exist for meniscal repair, and the choice of technique largely depends on tear morphology, location, and surgeon preference [1]. In the present study, the consistent use of a single all‐inside cerclage ('hay bale') repair technique across all cases reduced heterogeneity related to surgical technique, thereby strengthening the internal validity of the findings.

Future studies should evaluate the impact of PTS and SATT in larger cohorts to confirm or refute these preliminary findings. In addition, further investigations focusing on LM repair failure, ideally in larger cohorts, would provide valuable complementary insights.

This study has several limitations. First, the definition of MMRF was limited to cases requiring revision surgery and did not include patients with persistent symptoms, incomplete healing, or radiographic progression managed nonoperatively. Subgroup analyses in this study involved relatively small sample sizes, thereby reducing the statistical power and increasing the risk of type II error. Therefore, the results should be interpreted with caution, and studies with larger cohorts are needed to confirm these findings. Nevertheless, given the retrospective nature of this study, all available patients were included. The retrospective design is associated with inherent biases. Approximately 10% of patients were lost to follow‐up, which remains acceptable given the minimum 6‐year follow‐up and the young age of the study population. For those diagnosed with MMRF who did not return to our centre, clinical confirmation of the diagnosis was not possible. Exclusion of patients with concomitant graft rupture may have introduced selection bias and potentially attenuated the observed effect of PTS. However, this approach allowed us to isolate patients with an intact graft and to exclude MMRF occurring in the context of graft rupture or subsequent to graft rupture. Details regarding the exact type of meniscal tear were not available and subgroup analyses should be performed in future studies, even if tear length, type, or location did not appear to be associated with repair failure [42]. Finally, data on activity level, functional outcomes, coronal alignment, body mass index, meniscal extrusion, exact number of sutures and tear chronicity were not available, despite their potential influence on meniscal repair outcomes.

## CONCLUSION

No statistically significant association was detected between PTS, SATT, DATT, age or the presence of LET and MMRF after hamstring ACLR. However, larger studies are needed, particularly in high‐slope subgroups. After ACLR, PTS and SATT appear to be more strongly associated with graft‐related outcomes than with MMRF. Increased PTS or SATT alone should not discourage MM repair in the setting of ACLR.

## AUTHOR CONTRIBUTIONS

David Mazy, Nicolas Cance and Michael James Dan drafted the manuscript. David Mazy and David Henri Dejour were responsible for the research design. David Mazy, Nicolas Cance, Lucia Angelelli, Tomas Pineda, and Michael James Dan were responsible for data acquisition. David Mazy, Nicolas Cance, Lucia Angelelli, Tomas Pineda, Michael James Dan and David Henri Dejour analysed and interpreted the data. All authors reviewed and approved the final manuscript.

## CONFLICT OF INTEREST STATEMENT

David Henri Dejour has received royalties from Arthrex, Science & BioMaterials (SBM), and Corin; and consulting fees from Smith & Nephew. The other authors declare no conflict of interest.

## ETHICS STATEMENT

All patients provided informed consent for the use of their data for research. The study was approved by the institutional ethical board (No. COS‐RGDS‐2020‐03‐006‐DEJOUR‐D).

## Data Availability

The data that support the findings of this study are available on request from the corresponding author. The data are not publicly available due to privacy or ethical restrictions.
